# Does Diabetes Mellitus Increase the Short- and Long-Term Mortality in Patients With Critical Acute Myocardial Infarction? Results From American MIMIC-III and Chinese CIN Cohorts

**DOI:** 10.3389/fendo.2021.797049

**Published:** 2021-12-14

**Authors:** Shiqun Chen, Zhidong Huang, Liling Chen, Xiaoli Zhao, Yu Kang, Wenguang Lai, Xiaozhao Lu, Yang Zhou, Yibo He, Haozhang Huang, Qiang Li, Jin Liu, Yan Liang, Shaohong Dong, Ning Tan, Yong Liu, Jiyan Chen

**Affiliations:** ^1^ Department of Cardiology, Guangdong Provincial Key Laboratory of Coronary Heart Disease Prevention, Guangdong Cardiovascular Institute, Guangdong Provincial People’s Hospital, Guangdong Academy of Medical Sciences, Guangzhou, China; ^2^ Department of Cardiology, Longyan First Hospital Affiliated With Fujian Medical University, Longyan, China; ^3^ Department of Cardiology, The Third Affiliated Hospital of Sun Yat-Sen University, Guangzhou, China; ^4^ Department of Cardiology, the First People’s Hospital of Kashgar, Kashi, China; ^5^ Department of Cardiology, Maoming People’s Hospital, Maoming, China; ^6^ Department of Cardiology, Shenzhen People’s Hospital, Shenzhen, China

**Keywords:** acute myocardial infarction, critical, diabetes mellitus (DM), short-term mortality, long-term mortality

## Abstract

**Background:**

The harmful effect of diabetes mellitus (DM) on mortality in patients with acute myocardial infarction (AMI) remains controversial. Furthermore, few studies focused on critical AMI patients. We aimed to address whether DM increases short- and long-term mortality in this specific population.

**Methods:**

We analyzed AMI patients admitted into coronary care unit (CCU) with follow-up of ≥1 year from two cohorts (MIMIC-III, Medical Information Mart for Intensive Care III; CIN, Cardiorenal ImprovemeNt Registry) in the United States and China. Main outcome was mortality at 30-day and 1-year following hospitalization. Kaplan-Meier curves and Cox proportional hazards models were constructed to examine the impact of DM on mortality in critical AMI patients.

**Results:**

1774 critical AMI patients (mean age 69.3 ± 14.3 years, 46.1% had DM) were included from MIMIC-III and 3380 from the CIN cohort (mean age 62.2 ± 12.2 years, 29.3% had DM). In both cohorts, DM group was older and more prevalent in cardio-renal dysfunction than non-DM group. Controlling for confounders, DM group has a significantly higher 30-day mortality (adjusted odds ratio (aOR) (95% CI): 2.71 (1.99-3.73) in MIMIC-III; aOR (95% CI): 9.89 (5.81-17.87) in CIN), and increased 1-year mortality (adjusted hazard ratio (aHR) (95% CI): 1.91 (1.56-2.35) in MIMIC-III; aHR (95% CI): 2.62(1.99-3.45) in CIN) than non-DM group.

**Conclusions:**

Taking into account cardio-renal function, critical AMI patients with DM have a higher 30-day mortality and 1-year mortality than non-DM group in both cohorts. Further studies on prevention and management strategies for DM are needed for this population.

**Clinical Trial Registration:**

clinicaltrials.gov, NCT04407936.

## Introduction

The prevalence of diabetes mellitus (DM) ranges from 20-30% in acute myocardial infarction (AMI) patients (1, 2), and the burden of DM has been heavy and keeps increasing among AMI patients (3). The Thrombolysis in Myocardial Infarction (TIMI) study, a multi-center, maximus sample study, indicated that ST-segment elevation myocardial infarction (STEMI) patients with DM have a 40% higher 30-day mortality and 22% higher 1-year mortality than those without DM in a cohort of 62,036 patients from 55 countries (4). To date, however, numerous studies have observed no significant association between DM and short- or long-term mortality (5, 6). Recently, an Italian cohort study also reported DM is not significantly associated with short-term mortality in STEMI patients, possibly due to underlying cardio-renal dysfunction in patients with DM (7). Therefore, the independent harmful effect of DM on short- and long-term mortality in AMI patients may be inconsistent. Furthermore, most studies on the relationship between DM and prognosis were conducted among the unselected AMI population (5, 6), while limited studies focused on AMI patients first admitted to CCU that are more prone to hemodynamic instability, multiple organ dysfunction and sudden death.

To provide reliable evidence, we investigated the possible harmful effect of DM on short- and long-term mortality following critical AMI in the American Medical Information Mart for Intensive Care (MIMIC-III) cohort and Chinese Cardiorenal ImprovemeNt (CIN) cohort.

## Materials and Methods

### Data Sources and Study Population

We analyzed two existing datasets to examine the significance of DM on 30-day and 1-year mortality in patients with critical AMI. In the American cohort, we analyzed a coronary care unit (CCU) cohort from the MIMIC-III database describing CCU admissions to the Beth Israel Hospital (Boston MA, USA) from 2001 to 2012 (8). To access the database, we completed the National Institutes of Health’s web-based course Protecting Human Research Participants (certification number 36478705). In the Chinese cohort, we analyzed CCU admissions to an advanced teaching hospital, Guangdong Provincial People’s Hospital (Guangdong, China). The CIN study (ClinicalTrials.gov NCT04407936) enrolled consecutive patients undergoing coronary angiography (CAG) or percutaneous coronary intervention (PCI) from January 2007 to December 2018 in Guangdong Provincial People’s Hospital, Guangdong, China.

**Figure 1:**
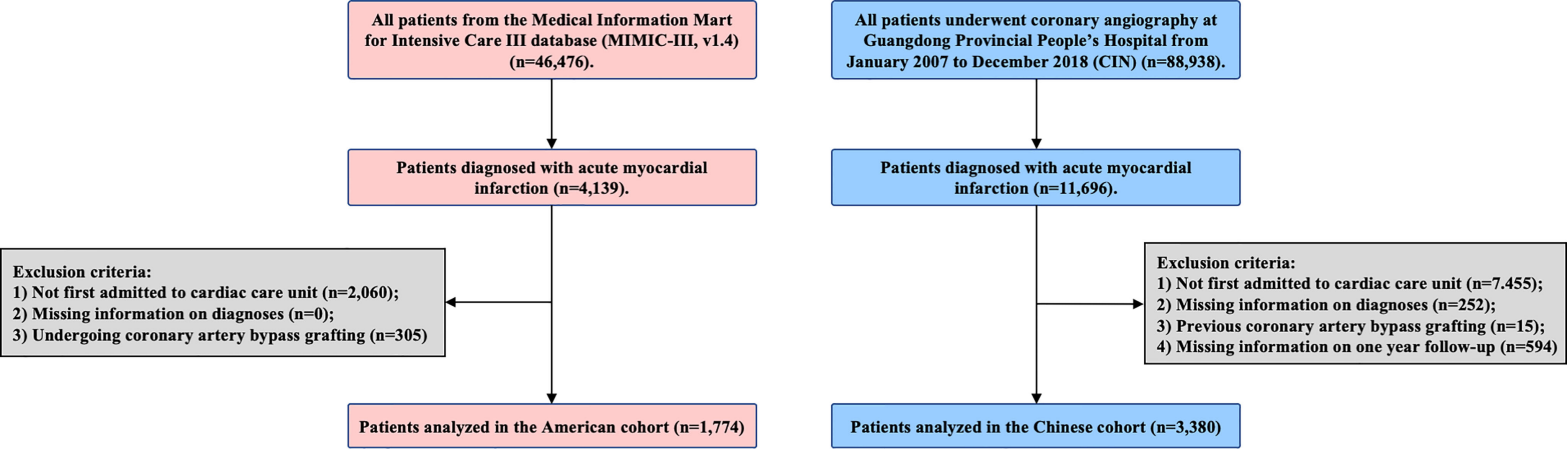
Patient flow diagram of MIMIC-III and CIN.

This study included patients with acute myocardial infarction (AMI) who had been admitted to the CCU on admission. Patients with missing information on diabetes diagnosis and cardiac and renal function were excluded. All patients were followed for at least 1 year. Eventually, 1774 patients were included in the MIMIC-III, and 3380 patients were included in the CIN (**Figure 1**). All traceable personal identifiers were removed from the analytic dataset to protect patients’ privacy. The study was approved by the local ethics committee and was performed according to the Declaration of Helsinki.

### Data Collection

MIMIC-III (1.4 version) database includes information from 2002 to 2011. Hourly physiologic readings from bedside monitors, validated by CCU nurses, were recorded. The database also contains records of demographics, laboratory results, and other clinical variables. International Classification of Diseases, Ninth Revision (ICD-9) codes was also documented for specific diseases by hospital staff on patient discharge. This information is extracted from the database based on Structure query language (SQL) (website: https://github.com/MIT-LCP/mimic-code). The follow-up was started from the date of admission and ended at death.

The data of CIN were extracted from the electronic clinical management records system of the Guangdong Provincial People’s Hospital. We examined all primary and secondary care records. The baseline information included demographic characteristics, coexisting conditions, laboratory examinations, and medications at discharge. The follow-up information was matched from the Guangdong Provincial Public Security based on the electronic Clinical Management System of the Guangdong Provincial People’s Hospital records.

### Endpoint and Variable Definition

The primary endpoint was 30-day and 1-year mortality. DM was diagnosed if this disease and/or antidiabetic treatment, including oral agents or insulin, were recorded in the medical record. Anemia was defined as a hematocrit ≤39% for males or ≤36% for females. The estimated glomerular filtration rate (eGFR) was calculated by the Modification of Diet in Renal Disease (MDRD) formula, and chronic kidney diseases (CKD) were defined as eGFR <60 mL/min/1.73m² (9). Congestive heart failure (CHF) was defined as New York Heart Association (NYHA) class > 2 or Killip class > 1 (10). Acute myocardial infarction (AMI), hypertension and atrial fibrillation (AF) were defined using ICD-10 codes.

### Statistical Analysis

Baseline characteristics were presented as means ± SDs for continuous variables, and proportions for categorical variables. The differences of baseline characteristics between groups were compared using Student t-test for continuous variables and chi-square tests for categorical variables. Time-to-event data among groups are presented graphically using Kaplan-Meier curves and compared by the log-rank test. In addition, 30-day and 1-year mortality were compared in critical AMI patients with or without DM in MIMIC-III and CIN cohort. Multivariable Logistic and Cox regression models were used to examine the relationship between DM and 30-day and 1-year mortality, respectively. The factors used to construct the Multivariable Logistic and Cox regression models in this study included: age, sex, hypertension, CKD, CHF, PCI, AF and anemia. Three models were sequentially constructed with or without adjustment for covariates: 1) unadjusted; 2) CHF and CKD; 3) age, sex, PCI, comorbidities (CHF, CKD, AF, hypertension and anemia). Presented tests were 2-tailed for all, and a p-value <0.05 was considered statistically significant. All statistical analyses were performed using R (ver. 4.0.3).

## Results

A total of 1774 patients (mean age 69.3 ± 14.3 years, 46.1% had DM) were included in the American cohort and 3380 (mean age 62.2 ± 12.2 years, 29.3% had DM) in the Chinese cohort. In the MIMIC-III cohort, 37.8% were female, 43.6% and 43.3% had CHF and CKD. In the CIN cohort, 19.6% were female, 24.0% and 25.0% suffered from CHF and CKD.

In both cohorts, DM group tended to be older than non-DM group. In the American cohort, no significant difference in DM prevalence was observed in gender, while female patients were more in DM group compared with non-DM group (26.4% vs. 16.8%) in the CIN cohort (p<0.001). In both cohorts, the prevalence of CKD and CHF was significantly higher in DM group than in non-DM group. Among patients with DM, the American cohort had a higher prevalence of CHF, CKD, and lower use of PCI than the CIN cohort at baseline. AMI patients undergoing PCI were less in DM group compared with non-DM group (90.5% vs. 92.7% in the CIN cohort; 64.06 vs. 75.73 in MIMIC-III cohort, respectively). More details of the baseline information of both cohorts are listed in **Table 1**.

**Table 1:** Baseline characteristics of critical AMI patients with and without DM in American MIMIC-III and Chinese CIN databases.

Characteristics	MIMIC-III (N=1774)			CIN (N=3380)		
	Non-DM	DM	P-value	Non-DM	DM	P-value
	N=956	N=818		N=2388	N=992	
**Demographic characteristics**						
Age, year	67.7 ± 15.3	71.1 ± 12.7	<0.001	61.4 ± 12.3	64.1 ± 11.5	<0.001
Female, n (%)	355 (37.13)	316 (38.63)	0.549	400 (16.75)	262 (26.41)	<0.001
**Complication**						
Hypertension, n (%)	450 (47.07)	376 (45.97)	0.676	1060 (44.39)	607 (61.19)	<0.001
CHF, n (%)	340 (35.56)	434 (53.06)	<0.001	484 (20.27)	327 (32.96)	<0.001
CKD, n (%)	290 (30.95)	463 (57.59)	<0.001	502 (21.02)	344 (34.68)	<0.001
Stroke, n (%)	24 (2.51)	50 (6.11)	<0.001	115 (4.82)	83 (8.37)	<0.001
PCI, n (%)	724 (75.73)	524 (64.06)	<0.001	2214 (92.71)	898 (90.52)	0.038
Anemia, n (%)	509 (54.32)	491 (61.15)	0.005	777 (34.83)	421 (44.32)	<0.001
AF, n (%)	192 (20.08)	217 (26.53)	0.002	46 (1.93)	27 (2.72)	0.187
**Medications**						
β blocker, n (%)	774 (80.96)	685 (83.74)	0.143	1993 (83.46)	723 (83.29)	0.954
Statins, n (%)	765 (80.02)	693 (84.72)	0.012	2260 (94.64)	815 (93.89)	0.462
ACEI or ARB, n (%)	673 (70.40)	566 (69.19)	0.618	1615 (67.63)	528 (60.83)	<0.001
DAPT, n (%)	729 (76.26)	642 (78.48)	0.289	2119 (88.74)	775 (89.29)	0.705

ACEI, angiotensin converting enzyme inhibitor; AF, atrial fibrillation; AMI, acute myocardial infarction; ARB, angiotensin receptor blocker; CHF, congestive hearts failure; CKD, chronic kidney disease; DAPT, dual antiplatelet therapy; PCI, percutaneous coronary intervention.

### Thirty-Day and 1-Year Mortality

**Figure 2:**
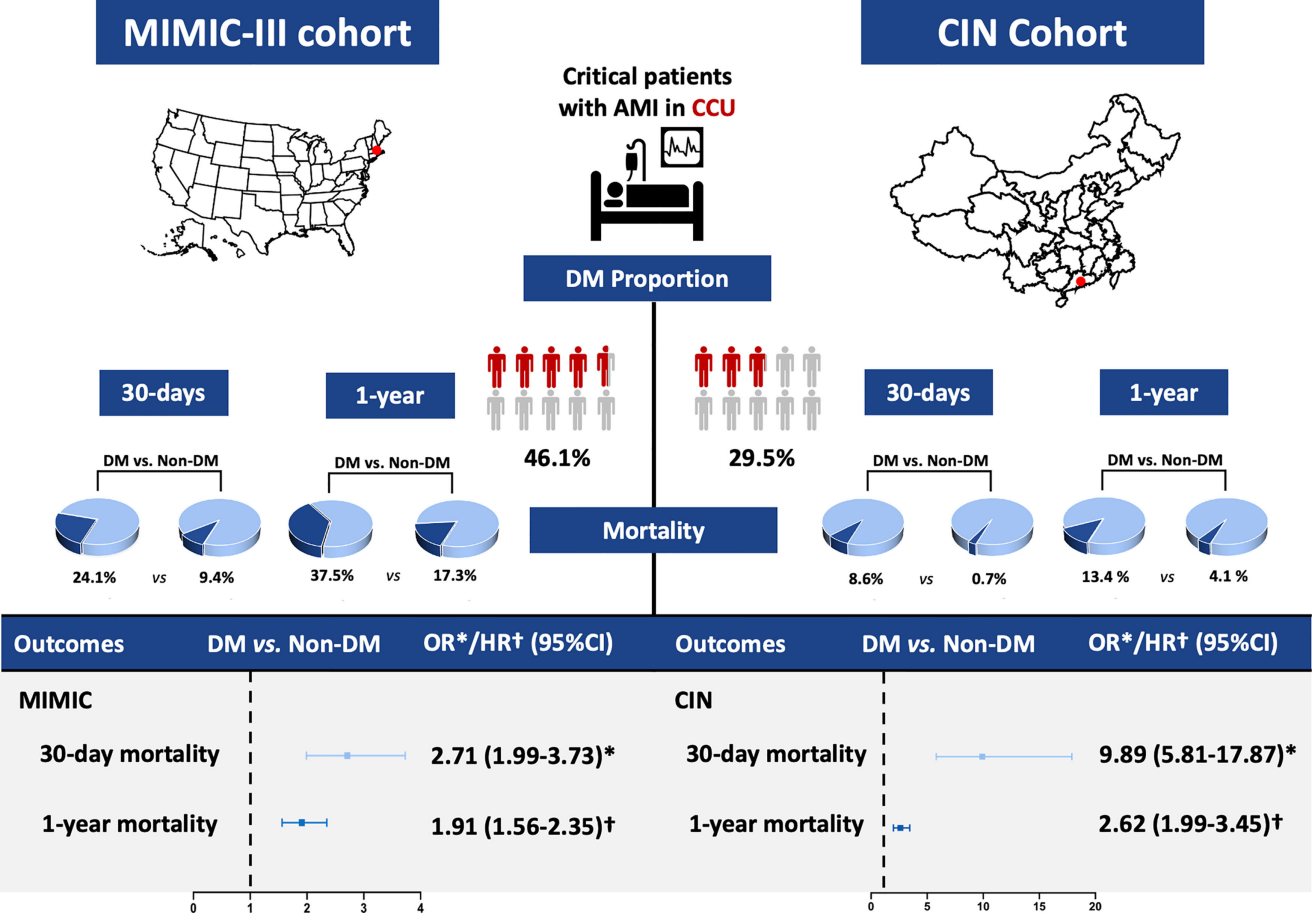
Summary graph of the role of DM in patients with critical AMI in the MIMIC-III and CIN cohort. *Odds ratio. ^†^Hazard ratio.

**Figure 3:**
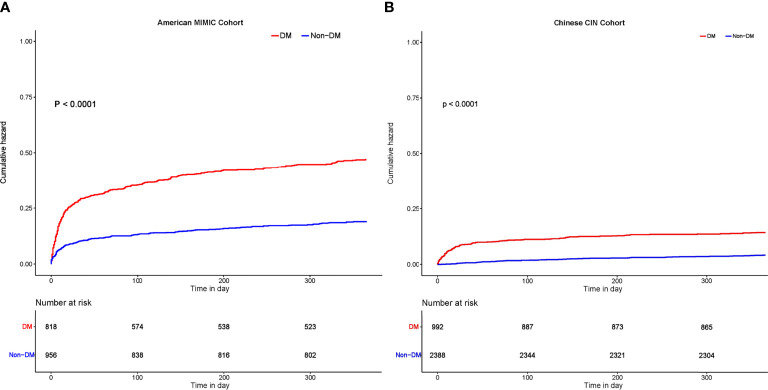
Kaplan-Meier curves for all-cause mortality in patients with critical AMI with and without DM. **(A)** 1-year mortality among patients with critical AMI with and without DM in MIMIC-III. **(B)** 1-year mortality among patients with critical AMI with and without DM in CIN.

At 30-day follow-up, a total of 288 patients (16.2%) in MIMIC-III cohort and 102 (3.0%) in CIN cohort died. At 1-year follow-up, 473 (26.7%) and 230 (6.8%) died in MIMIC-III and CIN cohort (**Figure 2**). As Kaplan-Meier curves showed (**Figure 3**), the short- and long-term mortality of DM in critical AMI patients was significantly higher compared to non-DM group in both MIMIC-III and CIN cohort (Log-rank test, p<0.0001). Controlling for confounding variables, DM group had a higher risk of 30-day mortality than non-DM group in both cohorts (adjusted odds ratio (OR) (95% CI): 2.71 (1.99-3.73) in MIMIC-III; aOR (95% CI): 9.89 (5.81-17.87) in CIN) (**Table 2**); and had a significantly higher 1-year mortality in both cohorts [(adjusted hazard ratio (aHR) (95% CI): 1.91 (1.56-2.35) in MIMIC-III; aHR (95% CI): 2.62 (1.99-3.45) in CIN)] (**Table 3**).

**Table 2:** Logistic regression analyses of DM to predict 30-day mortality in patients with critical AMI in American MIMIC-III and Chinese CIN databases.

Database		Model 1		Model 2		Model 3	
		OR (95% CI)	P-value	OR (95% CI)	P-value	OR (95% CI)	P-value
**MIMIC-III**	**DM vs. Non-DM**	3.07 (2.35-4.04)	<0.001	2.52 (1.87-3.44)	<0.001	2.71 (1.99-3.73)	<0.001
**CIN**		13.07 (7.93-22.87)	<0.001	10.15 (6.11-17.88)	<0.001	9.89 (5.81-17.87)	<0.001

Model 1: unadjusted.

Model 2: adjusted for chronic kidney disease, congestive hearts failure.

Model 3: adjusted for age, sex, hypertension, chronic kidney disease, congestive hearts failure, percutaneous coronary intervention, atrial fibrillation, anemia.

**Table 3:** Cox proportional hazards analyses of DM to predict 1-year mortality in patients with critical AMI in American MIMIC-III and Chinese CIN databases.

Database		Model 1		Model 2		Model 3	
		HR (95% CI)	P-value	HR (95% CI)	P-value	HR (95% CI)	P-value
**MIMIC-III**	**DM vs. Non-DM**	2.49 (2.06-3.01)	<0.001	1.82 (1.48-2.23)	<0.001	1.91 (1.56-2.35)	<0.001
**CIN**		3.55 (2.73-4.62)	<0.001	2.76 (2.12-3.61)	<0.001	2.62 (1.99-3.45)	<0.001

Model 1: unadjusted.

Model 2: adjusted for chronic kidney disease, congestive hearts failure.

Model 3: adjusted for age, sex, hypertension, chronic kidney disease, congestive hearts failure, percutaneous coronary intervention, atrial fibrillation, anemia.

## Discussion

To our knowledge, this is the first study to examine the harmful effect of DM on short- and long-term mortality simultaneously in critical AMI patients from American and Chinese cohorts. In our two cohorts, DM is very common among critical AMI patients (nearly half and one-third, respectively). Even after taking into account cardio-renal function, both the American and Chinese cohorts suggest that critical AMI patients with DM have 1.71- and 8.89-fold higher 30-day mortality, and 0.91- and 1.62-fold higher 1-year mortality than non-DM group, respectively. Accordingly, the death burden of DM in critical AMI patients is heavier in the Chinese cohort than in the American cohort.

Notably, the prevalence of DM is very high in patients with critical AMI. In this study analyzing an American cohort and a Chinese cohort, 46.1% and 29.3% respectively of patients with critical AMI had comorbid DM. Previously, Atman et al. have reported a high prevalence of DM (54%) in patients with critical AMI admitted into CCU in a 1,598 Qatar cohort (11). Possible explanations for the even higher prevalence compared with our two study cohorts could be due to the country itself has a high overall prevalence of diabetes in Qatar, and could be related to the highly prevalent consanguinity in marriages in the society (11). Similar to our results, in the INTERHEART study including 12,431 AMI patients admitted to CCU or equivalent cardiology ward from 52 countries, the 3,030 Chinese AMI participants had a lower rate of self-report DM at admission compared with 9,431 AMI patients in other countries (13% vs. 23%) (12). Noticeably, both the MIMIC-III cohort and CIN cohort were substantially higher in the prevalence of DM than the INTERHEART study (18.5%) among AMI patients (13), probably because the INTERHEART study relied only on a self-report history of DM, underestimating the DM prevalence in its study population. Another reason for the difference in the prevalence of DM is that the INTERHEART study was conducted from 1999 to 2003 and predated both MIMIC-III and CIN cohorts by years, and the burden of DM had increased rapidly throughout those years (14). As is shown above, despite the overall improvement in healthcare service and therapies throughout the years, DM co-morbidity remains high in AMI patients and substantially higher in critical AMI patients.

Noteworthily, the prevalence of DM in patients with critical AMI in China seems lower compared to the United States. The reason for this phenomenon may be that physicians in the United States and China have different diagnostic standards for diabetes. The National Glycohemoglobin Standardization Program standardized the vast majority of assays used in the United States in 2000 (15, 16), and an HbA_1c_ level ≥6.5% was later supplemented into the American diagnostic criteria for diabetes besides fasting and 2-hour blood glucose levels (17). However, in China, physicians have been using the 1999 World Health Organization criteria to diagnose DM until 2020, only utilizing a fasting blood glucose ≥7.0 and 2-hour blood glucose of ≥7.8 mmol/l. Since less severe cases were not recognized as DM under the stricter diagnostic criteria, the prevalence of DM could be underestimated in China. Meanwhile, it may explain why the mortality risk of DM in critical AMI patients seems relatively higher in China. Nonetheless, new cases of DM among adults significantly decreased from 2008 to 2018 in the US (18), while China is experiencing a rapid increase in the burden of DM (19, 20).

Another noticeable difference between two cohort are the 30-day and 1-year mortality, regardless of DM status. There are some reasons accounting for the higher mortality of patients in the MIMIC-III cohort. The patients were older, had higher prevalence of comorbidities and worse cardio-renal function. Moreover, PCI was an effective means of treatment for AMI, but the use of PCI was distinctly less than that in CIN cohort. In addition, the enrollment period differs between the two study cohorts (American registry vs. Chinese registry, 2001 to 2012 vs. 2007-2018, respectively), with the continuous improvement of medical technology, the survival rate of patients was improving.

DM remains a good prognostic predictor even in critical AMI patients who have a higher risk of death. Our study reported DM is associated with a nearly 2 to 9-fold higher risk of 30-day mortality and increased risk of 1-year mortality in patients with critical AMI. Few studies have investigated the prognostic effect of DM in critical AMI patients. Several studies evaluated the impact of DM after AMI on excess mortality and indicated that DM was associated with over 50% increase in short- and long-term mortality following unselected AMI, even after adjusting for confounders (5, 6, 21). However, some studies reported conflicting results. In a nationwide cohort of 2,018 DM and 19,547 Non-DM patients with a first hospitalized AMI in the Netherlands, DM was not independently associated with increased risk of 28-day mortality, but the result was not fully adjusted due to limited data, including other risk factors, comorbidity and treatment (22). In addition, Syed et al. reported that no difference was seen in the 1-year major cardiac event (MACE) or mortality between those with and without DM, who presented with AMI and were treated with drug-eluting stents, after adjusting co-morbid conditions, but the DM population still had a higher unadjusted mortality, although the sample size was relatively small (161 DM and 395 Non-DM patients) (23). Additionally, DM may be associated with mortality but not independent of other variables. Marenzi et al. reported that the increased in-hospital mortality of DM patients with STEMI was mainly driven by their underlying cardio-renal dysfunction in an Italy cohort (7). Consistent with another cohort of elderly adults with non-ST-elevation ACS (NSTEACS) from Italy (24), DM and hyperglycemia on admission were associated with higher mortality, mostly because of preexisting cardiovascular and renal damage. Furthermore, previous studies suggested that DM with concomitant renal insufficiency or cardiovascular co-morbidities independently or synergistically was related to an increase in worse outcomes after AMI (25, 26). Similarly, in two cohorts of our study, CKD and CHF were independent risk factors of mortality. Thus, DM may act as both an independent and synergistic factor to aggravating disease progression in patients with critical AMI.

Consistently, patients with DM have a greater atherosclerosis burden, with more diffuse and more multivessel coronary artery disease (25). Besides, patients with DM and AMI present more rapidly accumulate micro-and macrovascular complications, which may contribute to their worse outcomes (21). DM independently increases short- and long-term mortality in patients with AMI, and some pathophysiological mechanisms have been proposed to account for the adverse influence, including abnormalities in the endothelial, vascular smooth muscle cell, and platelet function; decreased bioavailability of nitric oxide; increased oxidative stress; pro-inflammatory/thrombotic state (27).

All findings based on two cohorts from the United States and China support that clinician need to pay specific attention to critical AMI patients who are diagnosed with DM. Early DM screening and treatment for critical AMI patients may be an effective means to reduce short-term deaths. Appropriate relaxation of the diagnostic criteria for DM may help distinguish potentially high-risk patients, so as to provide timely management and treatment for better ourcomes. In China, HbA1c levels should be incorporated in the diagnosis of DM according to the latest guidelines to better identify DM patients. Clinicians should be aware of effective glycemic control (recommended target <180 mg/dL while avoiding hypoglycemia) (28) and stress blood glucose monitoring during follow-up. In addition, comprehensive systematic care after the event is needed in order to improve survival in critical AMI patients, incorporating intensification of care at various levels: aggressive management of multiple cardiovascular risk factors, strengthening guideline-recommended treatments, and importantly, patients’ education and support (29). Moreover, a reduction of mortality in critical AMI patients with DM may be achieved through cardio- and renal-protective therapies (7). Recently, sodium-glucose cotransporter 2 inhibitors (SGLT2i) were shown to provide cardiovascular protection and prevent renal function deterioration, moreover, SGLT2i can also inhibit platelet activation and may act synergistically with anti-platelet therapies in the setting of acute myocardial infarction (30–32), and could be of clinical benefit to this vulnerable population. Further research is needed on the development of novel interventions to reduce mortality and improve survival quality in critical AMI patients with DM.

### Limitations

First, because the current study was the retrospective pattern, our inferences did not reflect direct causality, and some relevant information has not been collected completely. To make up for the disadvantage, we conducted the study simultaneously analyzing two existing high-quality cohorts and observed consistent effects, which to some extent enhanced the reliability of our results. Second, the enrollment period differs between the two study cohorts, which may potentially influence both the short- and long-term outcomes of the enrolled patients (American registry vs. Chinese registry, 2001 to 2012 vs. 2007-2018), and may contribute to the noticeable difference between mortality of two cohorts. Third, the baseline status of the patients with critical AMI in the two countries is different, but for this reason, the relationship between DM and prognosis that we have verified is more applicable among critical AMI patients with DM. Fourth, the diagnostic criteria for DM was different between two countries. But it still manages to reflect a high prevalence of DM, and DM was proven independently associated with mortality in each cohort. Lastly, we only explored all-cause mortality for a single endpoint and did not have a more comprehensive assessment of the prognosis of critical AMI patients with DM, because neither cohort registered specific information about cause of death and other follow-up information. Nonetheless, for these critical AMI patients, cardiogenic death is the main cause of death.

## Conclusion

Both American and Chinese cohorts suggest that DM is very common among critical AMI patients (nearly half and one-third, respectively). Even after taking into account cardio- and renal function, critical AMI patients with DM have 1.71- and 8.89- fold higher 30-day mortality, and 0.91- and 1.62-fold higher 1-year mortality than non-DM group in MIMIC and CIN cohorts, respectively. Further studies are needed on prevention and treatment strategies on DM among critical AMI patients with DM, especially in China.

## Data Availability Statement

The original contributions presented in the study are included in the article/supplementary material. Further inquiries can be directed to the corresponding author.

## Ethics Statement

The studies involving human participants were reviewed and approved by Guangdong Provincial People’s Hospital ethics committee. Written informed consent for participation was not required for this study in accordance with the national legislation and the institutional requirements.

## Author Contributions

The authors’ responsibilities were as follows—Research idea and study design: SC, ZH, LC, JL, and YL. Data acquisition: XZ, YK, WL, XL, YZ, YH, HH, QL, YL, NT, and JC. Data analysis/interpretation: JL and SC. Statistical analysis: ZH. Supervision and mentorship: YL. Writing guidance: YL. Each author contributed important intellectual content during manuscript drafting or revision and accepts accountability for the overall work by ensuring that questions on the accuracy or integrity of any portion of the work are appropriately investigated and resolved. The authors declare that there is no competing interest. All authors contributed to the article and approved the submitted version.

## Funding

This research was funded and supported by the National Key Research and Development Program of China, Grant (2016YFC1301202), The National Science Foundation of China (81500520, 82070360), Study on the function and mechanism of the potential target for early warning of cardiorenal syndrome after acute myocardial infarction based on transformism (DFJH201919), Natural Science Foundation of Guangdong Province General Project (2020A1515010940), and Guangdong Provincial Science and Technology Plan Project (2017B030314041). The funders had no role in the study design, data collection, and analysis, decision to publish, or preparation of the manuscript; the work was not funded by any industry sponsors.

## Conflict of Interest

The authors declare that the research was conducted in the absence of any commercial or financial relationships that could be construed as a potential conflict of interest.

## Publisher’s Note

All claims expressed in this article are solely those of the authors and do not necessarily represent those of their affiliated organizations, or those of the publisher, the editors and the reviewers. Any product that may be evaluated in this article, or claim that may be made by its manufacturer, is not guaranteed or endorsed by the publisher.

## References

[1] Ahmed B Davis HT Laskey WK. In-Hospital Mortality Among Patients With Type 2 Diabetes Mellitus and Acute Myocardial Infarction: Results From the National Inpatient Sample, 2000-2010. J Am Heart Assoc (2014) 3(4):e001090. doi: 10.1161/JAHA.114.001090 25158866 PMC4310403

[2] Arnold SV Lipska KJ Li Y McGuire DK Goyal A Spertus JA. Prevalence of Glucose Abnormalities Among Patients Presenting With an Acute Myocardial Infarction. Am Heart J (2014) 168(4):466–70.e1. doi: 10.1016/j.ahj.2014.06.023 25262255 PMC4180044

[3] Gandhi GY Roger VL Bailey KR Palumbo PJ Ransom JE Leibson CL. Temporal Trends in Prevalence of Diabetes Mellitus in a Population-Based Cohort of Incident Myocardial Infarction and Impact of Diabetes on Survival. Mayo Clin Proc (2006) 81(8):1034–40. doi: 10.4065/81.8.1034 16901026

[4] Donahoe SM Stewart GC McCabe CH Mohanavelu S Murphy SA Cannon CP. Diabetes and Mortality Following Acute Coronary Syndromes. JAMA (2007) 298(7):765–75. doi: 10.1001/jama.298.7.765 17699010

[5] Bauters C Lemesle G de Groote P Lamblin N. A Systematic Review and Meta-Regression of Temporal Trends in the Excess Mortality Associated With Diabetes Mellitus After Myocardial Infarction. Int J Cardiol (2016) 217:109–21. doi: 10.1016/j.ijcard.2016.04.182 27179900

[6] Gholap NN Achana FA Davies MJ Ray KK Gray L Khunti K. Long-Term Mortality After Acute Myocardial Infarction Among Individuals With and Without Diabetes: A Systematic Review and Meta-Analysis of Studies in the Post-Reperfusion Era. Diabetes Obes Metab (2017) 19(3):364–74. doi: 10.1111/dom.12827 27862801

[7] Marenzi G Cosentino N Genovese S Campodonico J De Metrio M Rondinelli M. Reduced Cardio-Renal Function Accounts for Most of the In-Hospital Morbidity and Mortality Risk Among Patients With Type 2 Diabetes Undergoing Primary Percutaneous Coronary Intervention for ST-Segment Elevation Myocardial Infarction. Diabetes Care (2019) 42(7):1305–11. doi: 10.2337/dc19-0047 31048409

[8] Johnson AE Pollard TJ Shen L Lehman LW Feng M Ghassemi M. MIMIC-III, a Freely Accessible Critical Care Database. Sci Data (2016) 3:160035. doi: 10.1038/sdata.2016.35 27219127 PMC4878278

[9] Aguiar-Souto P Ferrante G Del Furia F Barlis P Khurana R Di Mario C. Frequency and Predictors of Contrast-Induced Nephropathy After Angioplasty for Chronic Total Occlusions. Int J Cardiol (2010) 139(1):68–74. doi: 10.1016/j.ijcard.2008.10.006 19056138

[10] Mehran R Aymong ED Nikolsky E Lasic Z Iakovou I Fahy M. A Simple Risk Score for Prediction of Contrast-Induced Nephropathy After Percutaneous Coronary Intervention: Development and Initial Validation. J Am Coll Cardiol (2004) 44(7):1393–9. doi: 10.1016/j.jacc.2004.06.068 15464318

[11] El-Menyar AA Albinali HA Bener A Mohammed I Al Suwaidi J. Prevalence and Impact of Diabetes Mellitus in Patients With Acute Myocardial Infarction: A 10-Year Experience. Angiology (2009) 60(6):683–8. doi: 10.1177/0003319708328568 19098013

[12] Teo KK Liu L Chow CK Wang X Islam S Jiang L. Potentially Modifiable Risk Factors Associated With Myocardial Infarction in China: The INTERHEART China Study. Heart (2009) 95(22):1857–64. doi: 10.1136/hrt.2008.155796 19482846

[13] Yusuf S Hawken S Ounpuu S Dans T Avezum A Lanas F. Effect of Potentially Modifiable Risk Factors Associated With Myocardial Infarction in 52 Countries (the INTERHEART Study): Case-Control Study. Lancet (2004) 364(9438):937–52. doi: 10.1016/S0140-6736(04)17018-9 15364185

[14] Collaboration NCDRF. Worldwide Trends in Diabetes Since 1980: A Pooled Analysis of 751 Population-Based Studies With 4.4 Million Participants. Lancet (2016) 387(10027):1513–30. doi: 10.1016/S0140-6736(16)00618-8 PMC5081106 27061677

[15] Little RR Rohlfing CL Wiedmeyer HM Myers GL Sacks DB Goldstein DE. The National Glycohemoglobin Standardization Program: A Five-Year Progress Report. Clin Chem (2001) 47(11):1985–92. doi: 10.1016/S0009-9120(01)00279-X 11673367

[16] International Expert C. International Expert Committee Report on the Role of the A1C Assay in the Diagnosis of Diabetes. Diabetes Care (2009) 32(7):1327–34. doi: 10.2337/dc09-9033 PMC2699715 19502545

[17] American Diabetes A. Diagnosis and Classification of Diabetes Mellitus. Diabetes Care (2013) 36(Suppl 1):S67–74. doi: 10.2337/dc13-S067 PMC3537273 23264425

[18] Control CfD, Prevention. National Diabetes Statistics Report, 2020. Atlanta, GA: Centers for Disease Control and Prevention, US Department of Health and Human Services (2020) p. 12–5.

[19] Xu Y Wang L He J Bi Y Li M Wang T. Prevalence and Control of Diabetes in Chinese Adults. JAMA (2013) 310(9):948–59. doi: 10.1001/jama.2013.168118 24002281

[20] Liu M Liu SW Wang LJ Bai YM Zeng XY Guo HB. Burden of Diabetes, Hyperglycaemia in China From to 2016: Findings From the 1990 to 2016, Global Burden of Disease Study. Diabetes Metab (2019) 45(3):286–93. doi: 10.1016/j.diabet.2018.08.008 30196138

[21] Alabas OA Hall M Dondo TB Rutherford MJ Timmis AD Batin PD. Long-Term Excess Mortality Associated With Diabetes Following Acute Myocardial Infarction: A Population-Based Cohort Study. J Epidemiol Community Health (2017) 71(1):25–32. doi: 10.1136/jech-2016-207402 27307468

[22] Koek HL Soedamah-Muthu SS Kardaun JW Gevers E de Bruin A Reitsma JB. Short- and Long-Term Mortality After Acute Myocardial Infarction: Comparison of Patients With and Without Diabetes Mellitus. Eur J Epidemiol (2007) 22(12):883–8. doi: 10.1007/s10654-007-9191-5 PMC2190782 17926133

[23] Syed AI Ben-Dor I Li Y Collins SD Gonzalez MA Gaglia MA. Outcomes in Diabetic Versus Nondiabetic Patients Who Present With Acute Myocardial Infarction and Are Treated With Drug-Eluting Stents. Am J Cardiol (2010) 105(6):819–25. doi: 10.1016/j.amjcard.2009.11.010 20211325

[24] Savonitto S Morici N Cavallini C Antonicelli R Petronio AS Murena E. One-Year Mortality in Elderly Adults With Non-ST-Elevation Acute Coronary Syndrome: Effect of Diabetic Status and Admission Hyperglycemia. J Am Geriatr Soc (2014) 62(7):1297–303. doi: 10.1111/jgs.12900 24917216

[25] Kim CS Choi JS Park JW Bae EH Ma SK Jeong MH. Concomitant Renal Insufficiency and Diabetes Mellitus as Prognostic Factors for Acute Myocardial Infarction. Cardiovasc Diabetol (2011) 10:95. doi: 10.1186/1475-2840-10-95 22035298 PMC3225317

[26] Kvan E Pettersen KI Sandvik L Reikvam A Investigators IS. High Mortality in Diabetic Patients With Acute Myocardial Infarction: Cardiovascular Co-Morbidities Contribute Most to the High Risk. Int J Cardiol (2007) 121(2):184–8. doi: 10.1016/j.ijcard.2006.11.003 17184858

[27] Paneni F Beckman JA Creager MA Cosentino F. Diabetes and Vascular Disease: Pathophysiology, Clinical Consequences, and Medical Therapy: Part I. Eur Heart J (2013) 34(31):2436–43. doi: 10.1093/eurheartj/eht149 PMC3743069 23641007

[28] Amsterdam EA Wenger NK Brindis RG Casey DE Jr. Ganiats TG Holmes DR Jr.. 2014AHA/ACC Guideline for the Management of Patients With Non-ST-Elevation Acute Coronary Syndromes: A Report of the American College of Cardiology/American Heart Association Task Force on Practice Guidelines. J Am Coll Cardiol (2014) 64(24):e139–228. doi: 10.1016/j.jacc.2014.09.017 25260718

[29] Canadian Diabetes Association Clinical Practice Guidelines Expert C Tardif JC L'Allier PL Fitchett DH. Management of Acute Coronary Syndromes. Can J Diabetes (2013) 37(Suppl 1):S119–23. doi: 10.1016/j.jcjd.2013.01.034 24070931

[30] Radholm K Figtree G Perkovic V Solomon SD Mahaffey KW de Zeeuw D. Canagliflozin and Heart Failure in Type 2 Diabetes Mellitus: Results From the CANVAS Program. Circulation (2018) 138(5):458–68. doi: 10.1161/CIRCULATIONAHA.118.034222 PMC6075881 29526832

[31] Spigoni V Fantuzzi F Carubbi C Pozzi G Masselli E Gobbi G. Sodium-Glucose Cotransporter 2 Inhibitors Antagonize Lipotoxicity in Human Myeloid Angiogenic Cells and ADP-Dependent Activation in Human Platelets: Potential Relevance to Prevention of Cardiovascular Events. Cardiovasc Diabetol (2020) 19(1):46. doi: 10.1186/s12933-020-01016-5 32264868 PMC7140327

[32] Nusca A Tuccinardi D Pieralice S Giannone S Carpenito M Monte L. Platelet Effects of Anti-Diabetic Therapies: New Perspectives in the Management of Patients With Diabetes and Cardiovascular Disease. Front Pharmacol (2021) 12:670155. doi: 10.3389/fphar.2021.670155 34054542 PMC8149960

